# The Hospital. Nursing Section

**Published:** 1903-05-09

**Authors:** 


### The Hospital.

### Nursing Section.

Contributions for this Section of "THE HOSPITAL" should be addressed to the EDITOR, "THE HOSPITAL" NURSING SECTION, 28 & 29 Southampton Street, Strand, London, W.C.

No. 867.—VOL. XXXIV. *SATURDAY, MAY* 9, 1903.

### Notes on News from the Nursing World.

### DEATH OF A CRIMEAN NURSE.

WE regret to hear of the death, at the age of 83, of Mrs. Sarah Ann Newton, who in her early days was a colleague of Miss Florence Nightingale. She was one of the band of nurses who went out to the Crimea with Miss Stanley, sister of Dean Stanley, in order to join Miss Nightingale's staff. Mrs. Newton was well known in Chelsea, where she had resided for many years, and might frequently be seen enjoying the air in her bath-chair drawn by a Chelsea pensioner.

### RESIDENT DISTRICT NURSES FOR IRELAND.

THE Countess of Dudley has always taken an interest in nursing, and an appeal which she has made to enable her to establish a fund for the purpose of providing resident district nurses for the poorest and most congested parts of Ireland can scarcely fail to meet with substantial support. The urgent need of these nurses is shown in a striking manner by the statement of a recent visitor to Glengariff. At the time of the visit there was a severe epidemic of measles amongst the poor, many of whom live in mud cabins, the sufferers including upwards of 650 children as well as several adults. During the whole of the time there was not a single nurse in the parish, which extends seven miles; and however serious the cases happened to be the patients and their friends had to do the best they could for themselves. No nurse can, in fact, be got at Glengariff, except by sending to Cork. We trust that by means of Lady Dudley's fund this and similar districts may soon participate in the advantages of skilled nursing.

### NURSING AT JOHANNESBURG.

AT the usual fortnightly meeting of the Johannesburg Hospital Board last month it was stated that the nursing staff was insufficient, and the superintendent applied for eight more nurses. The accommodation is at present inadequate; and as the nurses strongly object to living in tents, it has been suggested that a vacant house in the neighbourhood should be taken for them. The patients, of whom 140 are white persons, are still nursed by religious, as well as by lay, sisters. The former keep to their own wards on the ground floor, superintend the catering and the preparation of food; and Sister Aloysius is in charge of the operation theatre. The lay sisters have charge of the upper floors. For the post of matron of the institution 250 applications have been received. King Edward VII.'s Convalescent Home is now in working order. It is designed specially to assist young men and others away from home who cannot obtain home comforts after serious operations or illness. The twenty-five patients will be looked after by a matron and ten nurses.

### THE BLOT IN THE DEPARTMENTAL REPORT.

THE proceedings at the Eastern Counties Poor Law Conference in Colchester were of considerable interest to nurses. The report of the Executive Committee having been adopted, Dr. J. A. Fraser, medical officer of Romford Workhouse, read a paper on "Nursing in Workhouses," in the course of which he referred to the recommendation of the use of the term "qualified nurse" for probationers who had completed one year's training in a union school and had gained their certificate. He held that this unfortunate title was the one blot in the whole report, for though, having an assuring sound, the title was a mere sham and imposition, and was likely to bring discredit upon Poor Law infirmaries as training schools for nurses. Dr. Fraser strengthened the force of his destructive criticism by suggesting as an alternative that a probationer after a year of training at a union school should go for a further two years' term at a major school, and might thus place herself on an equality with the nurse who was doing the full three years' course. His views were substantially endorsed by the conference; and though there was some difference of opinion on the point whether the superintendent nurse should be given complete control over the infirmary, the majority of the speakers were extremely favourable to the proposal. The importance of according to every board of guardians the right of separating the administration of the infirmary from that of the workhouse, was vigorously insisted upon.

### MEETING OF THE MIDWIVES BOARD.

THE Central Midwives Board met on Thursday last week, Dr. Champneys in the chair. A letter was read from the Local Government Board enclosing copies of a circular letter and memorandum on the subject of the Midwives Act, 1902, which they had sent to the councils of counties and county boroughs in England and Wales. A communication from the Derbyshire County Council urging the desirability of forming a rule requiring every midwife to report all cases of puerperal fever within her practice to the medical officer of health of the district in which the case occurs, and also to the local supervising authority, was referred to the committee dealing with the subject. Respecting a letter from the Secretary of Queen Charlotte's Hospital enclosing a petition praying for the recognition of the hospital's certificate as a sufficient qualification under section 2 of the Act, the Secretary was instructed to reply that the question of admission under section 2 in respect of "such other certificate as may be approved by the Central Midwives Board" could not be decided immediately, and that the memorial from Queen Charlotte's Hospital should have careful consideration. The Chairman and Secretary reported as to offices which they had inspected, and the matter was left in their hands. The question of the general standard of education to be required from candidates for examination was considered, and it was resolved that the examiners should have the power to reject any candidate not sufficiently instructed in elementary education. It was resolved that the meeting of the Board should not be open to the public or to reporters except in the event of a special vote of the Board, but that the Secretary should furnish a summary of the proceedings to the Press. Considerable progress was made in the drafting of rules under section 3, I., of the Act.

### THE PROBATIONER PROBLEM AT POPLAR.

It is a matter for regret that the managers of the Poplar and Stepney Sick Asylum have declined by a majority of one to rescind a resolution passed some time ago for the purpose of transferring the duty of appointing probationers from the matron to managers. That the plan has not answered well is proved by the constant changes at the asylum. At the last meeting, when the resolution was under consideration, a statement was made that "ragging" was going on among the nurses, but no evidence was given in support of it. If the managers do not feel entire confidence in the capacity and judgment of the matron, they should say so; but as a highly-trained nurse herself, she has a right to expect that she should be allowed to choose her probationers. As long as they are selected by the managers, she cannot possibly exercise full authority over them. Moreover, she must, in the nature of things be better able than the clerk of the Board—who acts for the managers—to decide whether the candidates who apply for admission to the training school are qualified to undertake the work. There would probably be no probationer problem to solve at Poplar if the matron possessed the usual power.

### AN OPENING IN WEST AFRICA.

It will be recollected that a few months ago a correspondent wrote to call attention to the great want of trained nurses in the mission stations on the West African coast. We hear from Southern Nigeria of a nurse who, though practically single-handed, is in charge of two small hospitals, dispenses medicine three times weekly, diagnoses cases, prescribes for patients, does minor surgery, and dresses wounds. She also carries on the Evangelistic part of the work. Her only helpers are three natives, and though she has often appealed for help, the response has always been "no offers." There is scope here for any enterprising nurse who takes her avocation seriously, and who can render assistance all round, though, of course, there should be a doctor to diagnose and prescribe. As to the climate, nothing more than a common-sense care of health is necessary for the fairly strong.

### IPSWICH NURSES' HOME.

The permanent home for the Ipswich nurses, which the Princess Christian will formally declare open next Saturday, is well worthy of the county town of Suffolk. By the addition of a new wing to the north side of the building formerly occupied as a private house by the late Mr. William Turner, 21 bedrooms have been secured. On the ground floor are the matron's sitting-room, nurses' dining and reading rooms, an apartment for probationers, a waiting-room, cloakrooms, and other offices. One of the features is the storeroom for district nurses. Here medical and surgical appliances, sheets, and other linen for free loan for use among the poor will always be in readiness. As our readers know, the home has been furnished throughout at the expense of the donor, and everything essential, with many things that are not, has been provided. At the rear is an isolation block, for the use of nurses returning from infectious cases, in which five bedrooms have been arranged. Miss Hunt, the energetic lady superintendent of the home, and her staff are certainly to be congratulated upon the possession of their comfortable and commodious new quarters.

### GLOUCESTER DISTRICT NURSING SOCIETY.

Although there is a trifling balance on the wrong side, the annual report of the Gloucester District Nursing Society is, on the whole, very satisfactory, and the speeches at the annual meeting were of a singularly encouraging character. The report, which mentions the federation of the Society with the Royal National Pension Fund for Nurses—a step we hope to see taken before long by all important nursing organisations—shows that the work of the nurses has materially increased, and that in 1902 637 people were nursed, 359 midwifery cases attended, and 22,754 visits paid. A feature of the accounts is that the Board of Guardians contributed £150. This is a handsome grant which contrasts very favourably with the penurious sums given elsewhere in a grudging manner. The Dean of Gloucester and several of the clergy testified in the warmest terms to the value of the work. Special mention was also made of the munificence of Mr. W. Long, a citizen of Gloucester, to whom the district nurses are indebted for the possession of their perfectly-equipped home; and an address delivered by Miss Amy Hughes, who observed that the Gloucester District Nursing Society was in existence before the Queen's Jubilee scheme came into being, elicited a vote of thanks from the meeting.

### COTTAGE NURSES AND THE MIDWIVES ACT.

The annual meeting of the Norfolk Nursing Federation has just been held. A reference is made in the report, which was adopted, to the passing of the Midwives Act. It is pointed out that by its provisions no nurse will be allowed to act as a midwife who has not obtained a certificate from the Central Midwives Board, though a period of grace is given with some restrictions till April 1st, 1910, to those who cannot obtain such a certificate. All the nurses of the Norfolk Federation who act as midwives are required to register under the Act. As to the effect which the Act will have upon the supply of cottage nurses, the committee of the Association judiciously reserve their opinion until it has been in operation for some little time. Lord Lindley, in the course of his remarks, expressed a hope that there may be a large class of country midwives certified under the Act who will not be above living in the cottages, and in the fulfilment of that hope he foresees an immense boon to the poor.

### BRITISH GYNÆCOLOGICAL SOCIETY.

The first examinations of the British Gynæcological Society for its maternity nursing and gynæcological nursing certificates will be held on June 4th in London, and, if a sufficient number of candidates present themselves from the surrounding districts, the written part of the examination will also be held simultaneously on the same day in Newcastle-on-Tyne and in Birmingham. The *viva voce* examination of all English candidates will be held in the afternoon of June 18th in London. Arrangements are being made for the holding of the written part of these examinations in future in other important local centres, so as to save the candidates a second journey to the Metropolis. It is also proposed, as soon as the want is shown, to hold the written examinations in local centres in Scotland and Ireland, and the *viva voce* examinations for such nurses in Edinburgh and Dublin respectively. Those who desire to become candidates for the June examinations should apply at once for the necessary application forms to the secretary of the society, Dr. Aarons, 14 Stratford Place, London, W.

### BRADFORD GUARDIANS AND NURSES.

The Bradford Board of Guardians deserve to be bracketed with the Chorlton Board. Not only was the accommodation at the Nurses' Home at Bradford increased last year to the extent of 16 bedrooms, but in reviewing the work of the Board for the twelve months the chairman was able to state that the staff of nurses averages one to each seven patients. In these circumstances the chairman is clearly justified in claiming for the Bradford Guardians that, while the inmates of the institution under their care are not inefficiently nursed, the nurses are not overworked. It speaks well for the business capacities of the Board that in spite of the increase in the number of nurses there was no necessity to augment the rates.

### THE CRISIS AT BRIGHTON.

At a concert given in aid of the Brighton District Nursing Association the chairman warned the company that the existence of the organisation was in danger owing to the lack of support given by the public. The staff of the Queen's nurses has been already reduced to six owing to the fact that in 1902 there was a deficit of more than £700; and unless more liberal help is soon afforded, it may be found impossible to maintain even these. Concerts and similar entertainments, projected by the good-natured and charitably disposed, are of assistance in bringing grist to the mill; but the urgent need of Brighton is a subscription list proportionate to its wealth and prosperity. The abandonment of the nursing organisation would be nothing short of a disgrace to the town.

### "A WORKING SUPERINTENDENT NURSE."

An unusual proposal was discussed at the last meeting of the Bedford Board of Guardians. The visiting committee had passed a resolution recommending that applications should be invited for the appointment of "a working superintendent nurse," and several guardians questioned the expediency of using the word "working." Mr. Hull said that it was a wide term, and that the applicants would not know what was expected of them. It was eventually decided to delete the word and to add "who will take an active part in the nursing." This is an improvement on the original suggestion, and was perhaps not unnecessary. All superintendent nurses are expected to work, but their responsibilities are not always identical. For this reason it is desirable that an advertisement inviting applications for a vacant post should define the duties as clearly as possible.

### NURSES' HOME FOR ROCHDALE.

It was recently decided to build a nurses' home at Rochdale as a memorial of the long reign of Queen Victoria. The site of the old grammar school has since been secured, plans of a Manchester firm of architects have been accepted, and the work will be commenced at once. The home is to be on a large scale, and will cost several thousand pounds. Most of the money required has, however, been already subscribed.

### PORTSMOUTH PARISH INFIRMARY.

The written and oral examinations of the nurses of the Portsmouth Parish Infirmary were held by Dr. Bryant, of Guy's Hospital, on April 23rd and May 2nd respectively. Thirty-two nurses presented themselves, and of this number 28 passed. Only five of these, however, were final examinations, the rest being second and first year. The examiner said that the nurses had passed a high-class examination, and were a credit to their training school.

### A START AT SANDWICH.

It has been decided to start a District Nursing Association at Sandwich, which has the reputation of being one of the most sleepy of the Cinque Ports. But the enthusiastic manner in which the proposal was taken up at a meeting of ladies in the Town Hall shows that, in one respect at all events, it is wide awake. Not only was a resolution to make an effort unanimously passed, but the sum of £100 was fixed as the minimum requisite to justify the engagement of a nurse.

### IN MEMORY OF MISS L. M. GORDON.

The touching sermon preached in the chapel of St. Thomas's Hospital on Sunday, March 29, by the Rev. G. Weigall, vicar of Holy Trinity, Lambeth, and hospitaller of St. Thomas's, in memory of Miss L. M. Gordon has been printed by request. Many old St. Thomas's nurses will be glad to know that a copy can be obtained on application to the chaplain at the hospital.

### SHORT ITEMS.

A dramatic performance in aid of the Victoria Memorial Cot, founded by the *Ladies' Field* in memory of Queen Victoria, in the Victoria Hospital for Children, Chelsea, will be given at the Royal Court Theatre, on Saturday, May 16th, at three o'clock.

### The Nursing Outlook.

" From magnanimity, all fear above;
From nobler recompense, above applause,
Which owes to man's short outlook all its charm."

### NURSING HOMES.

In the course of the change of the nurse from a religious sister to a professional woman, where she shall find her home and how she shall be housed has been a difficult subject. In the hospitals there are the tiny bedrooms with general sitting-rooms, and very restricted liberty; in some of the homes run for private profit there is every liberty, but also great discomfort and absence of all home feeling.

In London the first move towards a wiser arrangement was made some fifteen years ago, and a hostel was started for nurses which was unconnected with any institution and was merely on a paying basis and for the convenience of nurses as a professional body. The rooms were small and cheap; some of them held two beds. The feeding arrangements were also cheap and plain, and the "social" side of home life was not attempted. Then another nurses' home was started by "charity," and it was a fine building, and the restaurant and rest rooms were good. But it was run by an outside committee and has been a financial failure.

Then a lady who looked at nursing from its modern and professional side, started a residential club for nurses, where there was a smoking-room, and where nurses could have their male friends to dine and give them champagne if they liked; and then the public began to be scandalised. Practically you can place all nursing homes in one of these three classes: The cheap, shabby home, where a nurse can sleep, but where she certainly cannot "re-create" herself between cases; the institutional home, where the nurse is still a slave as it were—still on duty and under the rule of an arbitrary committee; the home which is free of all restrictions, and a place not only of rest but of refreshment, but which is also regarded with suspicion and dislike by the public. Do any of these three fulfil our ideal; and if not, what is the ideal home for a private nurse?

The worst of it is, that it is only the ideal nurse who is fit for the ideal home: a nurse who has had all initiative trained out of her in school and hospital, who is an obedient machine and all right so long as she is under the thumb of a doctor or matron, but who is useless in any position of responsibility calling for reasoning power, is probably only fitted for the institutional home. There she is told what to do, and she does it. She gets up at 7, no matter how tired she is; she drinks coffee for her supper, no matter how sleepless she is; she eschews theatres and concerts because the rule is bed at 10. All that is customary and commonplace she fulfils day after day, and the idea of forming an opinion or a plan for herself never enters her head. She is more of a machine than a woman.

Then the shabby-genteel home is fitted for the old-fashioned nurse who is too tired and too poor to strike out in any way for herself. She wants to save for the old age looming so close ahead, and she is too worn to care for concerts, and the smell of tobacco makes her sick. She is probably happiest if she is in one of the semi-religious institutions where there is a bond of sisterhood through all holding the same creed, where the church or chapel services offer some freedom from self and glimpses of the larger life, and where some provision is made for old age. When one thinks of Mildmay, the Church Army, St. John' the Divine, the Wesleyan deaconesses, or the Little Sisters of the Poor, one recognises that the bond between nursing and religion is still very strong. But the bond between nursing and science is also very strong, and the modern scientific nurse wants to go her own way, and to be free to live her own life. This is all very well when she is off duty and out of uniform. But if the nurse is no longer the servant of the chaplain, she is still the servant of the medical man, and we confess to the utmost dislike to seeing a nurse in uniform behaving like an ordinary woman. We believe a nursing uniform to be out of place in a theatre, and we believe a cigarette to be out of place in a nurse's mouth. And in the same way is it not a pity to have a club specially for nurses' use, but where the uses are unnurselike? Suppose a woman, who is also a nurse, holds with Hamlet that we were not given

"Capability and god-like reason,
To fust in us unus'd"

and who wants to go to theatres and lectures, who wants to travel and to talk and to experience all sides of life, would she not be better, between cases, to wear no uniform and to live in ordinary dwellings with women writers and others, and not to label herself "nurse" in any way? There has been something semi-religious about the nursing uniform, there is still something *dévote* about the career of a nurse; so long as this remains a home specially labelled for nurses should be careful not to offend against the strictest standard. On the other hand the narrowness of the nursing outlook would be considerably enlarged if the machine-made nurse could be sent to dwell amongst thinking, feeling women and taught that it is not sinful to have views of her own. The number of trained nurses is now so great that every class and type is necessarily to be met with. The time is approaching, if it has not already come, when each type will tend to gravitate to some centre, and many of the present difficulties, and much of the criticism which to day is unjustly levelled at nurses as a body, will be concentrated on those alone who lay themselves open to it. The evolution of the model type of trained nurse takes years, but before long practitioners and public will be able to obtain her services with certainty whenever they need them.

### Lectures on Medical and Surgical Nursing.

By John Hopkins, F.R.C.S., Medical Superintendent of the Central London Sick Asylum District Asylums, Cleveland Street, W., and Hendon, N.W.

### LECTURE VII.—RHEUMATISM AND GOUT.

*Acute Rheumatism* is a disease in which the joints are inflamed, and the pain is often severe. The temperature sometimes rises to a dangerous height. Inflammation of the pericardium and of the heart valves is a frequent complication. A sour-smelling perspiration, profuse and giving rise to a rash is characteristic of the disease. The rash is composed of sudamina, which are collections of perspiration in drops under the epidermis, and have a translucent glistening appearance, without any redness around them.

Relapses frequently occur and convalescence is slow. There are all degrees of rheumatism. When the inflammation of the joints is accompanied by a moderate elevation of temperature, the case is called subacute. There is little or no rise of temperature in many cases in which nevertheless there is some pain in one or more joints and a little puffiness around them from the effusion of a small quantity of fluid into the cavity of the joint.

In dealing with acute rheumatism, disturb the patient as little as possible. Avoid shaking the bed, for the slightest jar causes acute pain. When the pericardium contains fluid, and the cardiac valves are affected, the heart is at a great disadvantage; and to sit the patient up in such a condition may lead to serious consequences. The pains are relieved completely and the temperature reduced to normal by salicylate of soda in many cases, so that the patient and his friends think he is well and fit to get up; and it is sometimes necessary to stop the medicine to convince them that this is not the case; for as soon as the medicine is left off, the pains and the rise of temperature return. Before a patient can be pronounced well, it is necessary to discontinue the drug and to keep the patient under observation for a while. During recovery it must be borne in mind that the heart has been weakened when there has been inflammation about it and that perhaps it has to do more work than formerly whilst in this weakened state; for the valves shrink and allow of blood to flow backwards through them, so that at every beat of the heart there is work wasted and which must be done over again. So no great exertion must be sanctioned, until the heart has acquired the necessary strength.

*Arthritis deformans.*—You are all familiar with the great deformity that results from this disease, as the infirmary necessarily becomes the home of this class of cases among the very poor. It has other names, such as *rheumatoid arthritis*, *rheumatic gout* and *osteo-arthritis*. This disease may follow acute rheumatism or come on gradually. It is due to a degeneration of the cartilage covering the ends of the bones, whilst at the same time there is a new growth of cartilage round the edges of the articular cartilage, in the ligaments and in the synovial membrane. The synovial fringes that hang into the knee joint when they have this growth of cartilage in them, are apt to get nipped between the bones in walking, and this causes a sudden pain and may throw the patient down. When a cartilaginous body is torn off the synovial fringe in this way it becomes very troublesome, gets caught and wedged in between the bones, sprains the knee, and causes more and more damage to the joint with each occurrence. These loose cartilages are sometimes removed by operation. Occasionally the pains are severe in arthritis deformans, but in many of the milder forms and in some of the severest there is little complaint, except when the joints are moved.

Stiffness of the joints is a consequence of these changes in them, and the pain on attempted movement tends to keep them in one position. This in itself is enough to produce stiffness in time, as is seen when a limb has been fixed in a splint for a few weeks; for when this is removed the fingers, for instance, can only be moved after the bands that have formed and that bind the tendons down have been broken through by forcibly bending the fingers. In dealing with a case of arthritis deformans this should be borne in mind. Much of the rigidity may be prevented by judicious exercise or by passive movement of the joints. Some of the rigidity is due to muscular action owing to the pain caused on attempted movement, and sometimes this is considerable: treatment by hot baths will often restore much of the lost power for a time. Massage, too, often proves beneficial, having an effect like the baths of relieving the pain and so enabling the patient to use his muscles unrestrained by this. This condition of joint is sometimes seen in sclerosis of the spinal cord, and in other conditions in which the nerves supplying the joints, etc., are degenerated. But in these cases the patients have lost the sense of pain, so there is no rigidity, the joints being moved and used freely. Hence, instead of being stiff, bent and painful, they are abnormally loose, flail-like and painless. This is then known as *Charcot's disease.*

*Gout* is an acute disease in its first onset, generally attacking the big toe joint only to begin with. It comes on about once a year, as a rule in the spring time, awaking the patient in the small hours of the morning. It tends to affect more and more joints, to come on more frequently, but to be less severe with lapse of time.

The inflammation is due to a deposit of chalky-looking material in the joints, composed of urate of soda. Uric acid is but slightly soluble, and when existing in excess in the blood gets deposited in the tissues about the joints and in other places. In the ear it forms the well-known chalk stones.

The subjects of gout are of irritable temper, and when suffering from an acute attack in which the pain is very severe, need very tender handling. Their diet needs to be regulated, all articles of food tending to increase the quantity of uric acid in the blood being avoided, such as beer and wines, meat, game, etc.

The attack of gout is the climax of a condition that has existed from birth perhaps, or at all events for some years before its onset. People in this condition are said to be lithæmic, from a word which means stone, lithic acid which is another name for uric acid, being the principal constituent of many stones formed in the kidneys and urinary bladder. Being but slightly soluble this substance becomes deposited not only in the tissues but in the urinary passages as well. As fine sand it is sometimes voided in considerable quantity and is known then as gravel. Those who are in this condition are often dyspeptic, irritable, and subject to fits of depression, alternating with high spirits and great energy. As children they are precocious and often subject to eczema. In advancing years there is a tendency to early degeneration of the arteries and to kidney disease. Death often results from cerebral hæmorrhage, due to the diseased blood-vessels giving way or becoming thrombosed.

In dealing with the subjects of lithæmia, the food should be plain and meat moderate in quantity. All alcoholic drinks should be avoided, and as much time as possible be spent in the open air.

### The Feeding of a Patient Suffering from Enteric Fever.

EXAMINATION QUESTIONS FOR NURSES IN THE COLONIES AND ABROAD GENERALLY.

THE question was as follows:—How would you feed a patient suffering from enteric fever, whilst the nourishment ordered by the doctor is to be strictly liquid. N.B.—The question supposes that the medical man in attendance gives only general orders and leaves the nurse to exercise her discretion.

FIRST PRIZE.

As enteric fever is a disease essentially affecting and diminishing the power of the whole alimentary tract, and as it must run a definite course involving a certain number of weeks, I should aim chiefly at giving the patient his nourishment in such a way as would best tend to its digestion and assimilation, and in such a manner as would alleviate the monotony of a liquid dietary.

Assuming that this consists of milk, I should (especially in the tropics where the cleanliness of vessels handled by natives is questionable) have it boiled, while broths, beef-tea, rice or barley-water would be carefully strained.

As a patient soon learns the art of rousing, even from deep sleep, and dozing again when fed at regularly recurrent intervals, five ounces of milk, his staple nourishment might be given two-hourly, making a total, irrespective of diluents, of three pints in the 24 hours. The broths and beef-tea, rice or barley-water being more readily absorbed could be given between these "feeds" reserving some brandy (if ordered) for the early morning.

In the tropics, where a distaste for milk is frequently encountered, where intense thirst, apart from fever, is induced, and where the digestive powers have been weakened by climatic conditions, I should endeavour to gain the physician's sanction to the following departures from recognised routine treatment:—

1. Permission to give the milk just lukewarm, and it must not be a fraction more than this, as constant and careful inspection of the dejecta has proved that as much as five pints per diem may thus be absorbed, while in the stools of patients fed upon cold or iced milk, curds at some stage or other of the disease were almost invariably detected.

2. The introduction into the milk of some flavouring essence such as vanilla (usually much appreciated), or in uncomplicated cases, freshly-made tea or coffee, especially during early convalescence, when the sense of taste is returning and the invalid realises more fully the sameness of his menu.

3. Permission to give in a case of insatiable thirst a daily rectal injection of warm water, the patient being instructed to retain it.

Well-iced water is usually allowed in unlimited quantities in the tropics, and forms a grateful change to the lukewarm fluids.

Lastly, as in the supine position, if the head be slightly raised, small quantities can be given to a sick person quite as conveniently through the medium of a dainty cup or glass as through the inevitable "feeder," I should strive to lessen the monotony of a liquid diet by these means, cautioning him to sip rather than gulp a draught of milk—even if distasteful—as in the latter case it not infrequently curdles in the stomach and is productive of discomfort if not pain.

NINA.

SECOND PRIZE.

An adult suffering from enteric and at the liquid diet stage should have at least from two and a-half to three pints of milk in 24 hours. Eight ounces are given every three hours, and in between while broth, soup, fruit drinks, rice and barley-water as well as plenty of plain filtered water. The milk may be given plain or in the form of cocoa, coffee, tea, etc. If it is found that the milk is not being properly digested by the patient, it must be diluted, equal parts milk and water or barley-water, or it may require to be diluted even more. Should the milk not be digested even when diluted, it must be peptonised.

If the patient becomes very distended without other serious symptoms occurring, stop the milk for a time, 24 to 48 hours, and give more broth. On the other hand, if the patient has a tendency to diarrhœa, stop giving broth and soup until the diarrhœa ceases. MELBOURNE.

MERITS AND DEFECTS OF THE PAPERS GENERALLY.

It is interesting to see that these resemble greatly those in the papers of nurses at home.

"Nina's" answer is far in advance of others; this lady was successful last October. She shows considerable powers of observation, as when she mentions the ease with which a patient is roused and yet falls asleep again if the rousing comes usually at the same hours. Her plan of a daily rectal injection of warm water is also excellent, but I hope she will enlarge her views as to variety and give some attention (a matter too much neglected) to the question of copious drinks, other than nourishment pure and simple.

"Melbourne's" quantity of milk is rather scanty. Three pints is the smallest amount permissible at the period when it forms the staple food.

HONOURABLE MENTION.

This is gained by "Sister Edris," "Noiram," and "Donnybrook." "Donnybrook," whose paper is otherwise good, makes a mistake in giving so much as half-a-pint every two hours. Very few patients could assimilate that amount. She shows, however, considerable ingenuity in making use of materials at hand. "Sister Edris" should not make out a list of food. The small space at our disposal necessitates an answer (if it is to be printed) being plainly written.

QUESTION FOR OCTOBER NEXT.

State what precautions you would take for the prevention of bed-sores? N.B.—Do not enter on the subject of curative treatment, but confine yourselves strictly to prevention.

RULES.

The competition is open to all. Answers must not exceed 500 words, and be written on one side of the paper only. The pseudonym, as well as the proper name and address, must be written on the *same* paper, and *not on a separate sheet*. Papers may be sent in for fifteen days only from the day of the publication of the question. All illustrations strictly prohibited. Failure to comply with these rules will disqualify the candidate for competition. Prizes will be awarded for the two best answers. Papers to be sent to "The Editor," with "Examination" written on the left-hand corner of the envelope.

N.B.—The decision of the examiners is final, and no correspondence on the subject can be entertained.

In addition to two prizes honourable mention cards will be awarded to those who have sent in exceptionally good papers.

Note that, instead of the sentence referring to the competition being open for 15 days, all papers for the October competition must reach this office not later than September 20th. THE EXAMINER.

### Presentations.

GLASGOW ROYAL INFIRMARY.—Miss Jamieson, charge nurse at Glasgow Royal Infirmary, has been presented with a case of toilet requisites by the assistant nurses, past and present, on the occasion of her resignation. In making the presentation, Miss Anderson alluded to the pleasant relations which had always subsisted between Nurse Jamieson and her assistants, and wished her every success in her new undertaking.

HOSPITAL AND HOME FOR INCURABLE CHILDREN, MAIDA VALE.—Mrs. Bruce, on leaving the Hospital and Home for Incurable Children in Maida Vale, of which she has been matron, was presented with an illuminated address, a cheque by the committee, a gold enamel and pearl necklace from the ladies associated with her in her work, and with gifts from the nursing and domestic staff.

### The Midwives Act, 1902.

THE Local Government Board have issued a memorandum to County and County Borough Councils. In this document they commence by explaining that the Act came into operation on April 1st, 1903, and that its object is to secure the better training and supervision of midwives. The Act makes provision for the institution of a roll which will contain the names of midwives certified under the Act, and for the formation of a body to be called the Central Midwives Board, which will possess jurisdiction in regard to the issue of certificates and the admission to the roll of midwives, and will exercise a general control over the practice of such persons. The Central Board has been constituted, and consists of the following members:—W. J. Sinclair, M.D.; Miss J. Wilson; F. H. Champneys, M.D.; J. W. Cousins, F.R.C S.; F. P. Young, M.R.C.S., L.S.A.; C. J. Cullingworth, M.D., F.R C.P.; J. H. Johnstone, M.P.; Miss R. Paget; and Miss D. Oldham. Secretary, G. W. Duncan. The temporary office of the Board is at the Privy Council Office, Whitehall, S.W. The Board will publish annually a roll of midwives who have been duly certified under the Act, and no woman not certified will be termed "midwife" as used in the Act.

The local supervision of midwives is entrusted to the council of every county or county borough throughout England and Wales, who are thus made the local supervising authority over midwives in their respective areas. They will have certain specified duties. First, to give due notice of the effect of the Act to persons at present using the title of midwife (it is understood that the Central Midwives' Board contemplate preparing a form of notice for the use of local authorities to exercise general supervision over all midwives practising in their area) to investigate charges of malpractice, negligence, and misconduct, and if needful report to the Central Board; to suspend any midwife from practice if such suspension seem desirable; to supply to the secretary of the Central Board every January the names and addresses of all midwives who during the preceding year have notified their intention of practising; to keep a current copy of the roll of midwives for public inspection; and to report at once the death of any midwife, or a change in name or address of a midwife within the area.

The local supervising authority may delegate any powers or duties to a committee appointed by them, and women are eligible to serve on such committees. The local supervising authority may also delegate powers or duties to any district council within the area of the county, and women may also serve on these district councils. Every woman who is certified as a midwife under the Act before holding herself out as a practising midwife, or commencing to practise as a midwife in any area, must give notice in writing of her intention to do so to the local supervising authority or the committee or district council appointed by them, and a like notice in the January of every year as long as she wishes to practise. This notice must be given to the local supervising authority of the area in which the woman resides or carries on her practice. If at any time she acts in another area as midwife, notice must also be given to the local supervising authority of that area within forty-eight hours at the latest from the time she commences to practise.

Every notice must contain such particulars as will secure the identification of the person giving it. If any woman omit to give the necessary notices or knowingly and wilfully make a false statement, she will be liable to a penalty not exceeding £5. If, after April, 1905, any woman not certified under the Act shall take or use the title of midwife or imply that she is a person specially qualified to practise midwifery, she shall be liable to a fine not exceeding £5. Also, after April 1st, 1910, no woman shall habitually and for gain attend women in childbirth, otherwise than under the direction of a qualified medical practitioner, unless she is certified under the Act; and any woman so acting without being certified shall be liable to a fine not exceeding £10. Women who present themselves for examination or certificates as midwives to the Central Midwives Board shall pay certain fees. These fees will be devoted to the payment of expenses connected with the examination and certificate.

### TRAVEL NOTES AND QUERIES.

OSTEND—ROOMS WITHOUT BOARD (Ignorance).—Are you aware that Ostend is a very expensive place? First your journey; the cheapest way is by the General Steam Navigation Company, from Tower Bridge, first return 10s. 6d. I strongly dissuade you from taking rooms, they are very dear, and you are never at the end of your expenses. I am a good French scholar myself, but I should hesitate to embark on the plan. If, as you say, expense is a serious consideration, you should *not* go to Ostend, but if it is necessary, I should advise a cheap hotel. I can speak very well of the Café Royal, where you will have pension terms for seven francs a day, or make an arrangement with Mr. Schmid to omit lunch as you will be often out, and take you for less.

ST. SERVAN AND BAY OF ST. MALO (M. A. N.).—First class return, London to St. Malo, £2 13s. 8d.; second return, £2 1s. 2d. The latter quite comfortable. Pensions at St. Servan Maison Mathias, seven francs in the season, less at other times; also Miss Humfrey, 20 Place Constantine, the same terms. Pension, Primavere Rue Ville Pepin. At St. Enogat, across the Bay and adjoining Dinard Hôtel de St. Enogat, from six to seven francs. St. Servan, though not so pretty, is a better centre.

### A FORTNIGHT IN SWITZERLAND.

We shall start on Tuesday, August 4 h. The route will be *viâ* Dover-Calais (or Folkestone-Boulogne) direct to Berne. Thence to Interlaken and Wengen. We shall arrive at Wengen on Wednesday, and stay there for six or more nights. On the following Tuesday we shall go to Grindelwald, and those who wish to do so will stay there the rest of the time, starting homewards on Monday, August 17th. The return route will be *viâ* Lake Brienz, over the Brünig to Lucerne, and back through Paris.

Wengen is 4,000 feet above sea-level, commands a splendid view of the snow-capped Jungfrau, and is the centre of some of the most beautiful scenery in Switzerland. The return route, too, is one of exceptional beauty.

*Cost.*—Ten guineas for the fortnight, including second-class travelling (third on the mountain railway from Interlaken to Wengen and back), and board and lodging.

The tickets will be available for 25 days, so that those who wish to prolong their holiday can do so at Lucerne, Paris, and other places on the route.

I should like names to be sent to me as soon as possible, as Switzerland is very crowded during August, and the hotels have intimated to me that they must know before the end of June how many of us to expect.

Most of the rooms contain two beds, but I can obtain a few single rooms for those who care to pay another 12s.

An extra sum of 10s. must be charged to those who would like to spend half the time at Grindelwald as suggested above.

I shall be assisted by Miss Bradbrook, of Queen Margaret's School, Scarborough, and another colleague. One will return at the end of the fortnight with any who desire to be conducted on the homeward journey. I should prefer all the members of the party to be over 18 years of age.

### UNIVERSITY COURSE IN FRENCH AT NEUCHATEL.

Those who are taking part of the course beginning on August 12th can arrange for considerably less than the above sum, to travel out as members of my party and spend the intervening days at Interlaken or some other place near. They will get the advantage of the return route described above.

Further details will be sent on application.

L. EDNA WALTER, B.Sc., A.C.O.I.,

Inspector under Board of Education, Secondary Branch.

38 Woodberry Grove, Finsbury Park, London, N.

### Everybody's Opinion.

[Correspondence on all subjects is invited, but we cannot in any way be responsible for the opinions expressed by our correspondents. No communication can be entertained if the name and address of the correspondent are not given as a guarantee of good faith, but not necessarily for publication. All correspondents should write on one side of the paper only.]

### UNANSWERED LETTERS.

"A NURSE" writes: I should like to call your attention to what is probably only an act of forgetfulness, but which entails inconvenience on others. Being in need of a situation I answered two advertisements in your columns. In each case I enclosed a stamp for a reply. This was over a fortnight ago, and as yet none has come to either letter. The culprit in one case is a medical man; the other is a nurse, who, knowing as she must what seeking work means, should be more thoughtful than to keep a fellow nurse in suspense. Perhaps the publication of these instances will make others more careful.

### THE NURSE IN PRISON.

DR. H. B. DONKIN, one of his Majesty's Commissioners of Prisons, writes from the Prison Commission, Home Office, Whitehall, S.W.: In consequence of an article entitled "The Nurse in Prison," which appeared in THE HOSPITAL of April 25th, I have received a very large number of letters which it is impossible for me to answer. Having heard nothing of this article until my attention was called to it yesterday, and yet being desirous that the writers of the above-mentioned letters should not be misled in this matter, I ask you to be good enough to insert this letter in your next issue. There are practically no special appointments for nurses *as such* in the prison service. Nurses are selected from among the general body of prison officers according to their special fitness. All female officers are required to enter the service on the same basis, and to undergo the same general training. At the large prisons where there are always numerous inmates of the hospital there are, of course, several officers whose duty is almost entirely nursing. The initial appointment to the prison service is in the grade of assistant warders—salary from £45, rising to £50. On promotion to the rank of warder the salary is £55, rising to £70; principal warder, £70 to £80; chief warder, £100 to £120; matron, £80 to £180, according to grade. Nearly all female officers lodge in the prison, but are not supplied with board. Candidates with qualifications as nurses are regarded as desirable.

### THE MATRON WHO COULD NOT KEEP BOOKS.

"MATRON" writes: Referring to your remarks in the issue of April 25th as to "The Matron who could not Keep Books," may I express my entire sympathy with the matron of the Guildford, Godalming, and Woking Joint Isolation Hospital Board, whose services that Board have most unwarrantably dispensed with because she was not a bookkeeper. I cannot see why a matron should be supposed to be an expert accountant, which she must needs be if an accurate record has to be kept by her of every meal given to a patient, the receipt of all provisions and necessaries and disposal of same between patients and officers, and the checking of the stores, which the Local Government Board through their auditors so much desire. If a matron possesses all the requisite qualifications of an efficient administrator and superintendent of the nursing staff, is not this sufficient? In my case, the Joint Hospital Board under whom I have the pleasure to serve, had the sense and courtesy to recognise this by appointing a "Matron's Clerical Assistant" who takes from me the onerous burden of having to keep intricate accounts, for which I in common with matrons generally had had no previous training, and which ought always to lie outside the sphere of a matron's duties.

[How can a hospital matron be efficient who cannot keep and check the books appertaining to her office?—ED. THE HOSPITAL.]

### Appointments.

ACCIDENT HOME, HEDNESFORD, STAFFORDSHIRE.—Miss Annie Beetlestone has been appointed matron. She was trained at the Queen's Hospital, Birmingham, and has since been sister-in-charge in the same institution.

BELFAST HOSPITAL, TRANSVAAL.—Miss M. B. Horswell has been appointed matron. She was trained at Salisbury Infirmary, where she was afterwards sister. She has also been sister at the Suffolk General Hospital, the Taunton and Somerset Hospital, and matron of the Burgher Camp Hospital, Klerksdorp.

BOROUGH SANATORIUM, FOLKESTONE.—Miss N. Feasdale has been appointed staff nurse. She was trained at the City Hospital, Leeds, and has since been nurse at Croydon Borough Hospital and Dorchester Sanitary Hospital.

CANTERBURY BOROUGH ASYLUM.—Miss A. Thompson has been appointed matron. She was trained at Wharncliffe Asylum, Oxford.

COLDASH HOSPITAL, NEWBURY.—Miss Agnes Finch has been appointed staff nurse. She was trained at the Evelina Hospital for Children, Southwark, S.E.

PONTYPRIDD UNION.—Miss H. Hilton has been appointed charge nurse. She was trained at Wolverhampton Workhouse Infirmary, where she was afterwards charge nurse. She has since been night charge nurse at Bristol Workhouse Infirmary.

PRESTWICH INFIRMARY.—Miss Laura S. Withers has been appointed nurse. She was trained at the Infirmary, Dereham Road, Norwich, and has since held the post of nurse at the Union Hospital, Chorley, Lancashire.

ROSEHILL CHILDREN'S HOSPITAL, TORQUAY.—Miss A. Isabel Dalzell has been appointed matron. She was trained at the Queen's Hospital, Birmingham; was afterwards sister at the Royal Orthopædic and Spinal Hospital, Birmingham; took holiday duty for four months at the Birmingham and Midland Hospital for Women; was matron of Dr. Savage's Private Hospital, Birmingham, for two and a-half years; matron of the Hospital, Castle Douglas, N.B., for two years; has twice acted as temporary matron of the Rosehill Children's Hospital, Torquay; also twice as temporary matron of the North Riding Infirmary, Middlesboro; and, during the intervals between her appointments, has done a good deal of Private nursing.

ROYAL HALIFAX INFIRMARY.—Miss Mabel A. Lloyd and Miss Elizabeth Armstrong have been appointed sisters. Miss Lloyd was trained at the Royal Infirmary, Edinburgh, and has since been private nurse at Leeds Trained Nurses' Institution, and night sister at Grantham Hospital. Miss Armstrong was trained at the Western Infirmary, Glasgow, where she has since been charge nurse.

ST. MARY ISLINGTON INFIRMARY, HIGHGATE HILL.—Miss Lance Richards has been appointed sister. She was trained at Lewisham Infirmary, and has since been charge nurse at Brook Fever Hospital, London.

STEYNING UNION.—Miss Kate Harris has been appointed assistant nurse. She was trained at Wandsworth and Clapham Infirmary, and at the Western Hospital, Fulham. She holds the L.O.S. certificate.

WHITECHAPEL UNION INFIRMARY. — Miss Linda R Masters has been appointed midwife. She was trained at the Whitechapel Infirmary, and has since done staff nurse's duties for four years. She holds the L.O.S. certificate.

### "The Hospital" Convalescent Fund.

THE Hon. Sec. begs to acknowledge with thanks the receipt of 4s. 6d. from Miss L. B. Marsh, and of 2s. 6d. from Miss A. M. Hill; also of 6s. per the "Travel" Editor which was omitted to be acknowledged during March.

### Echoes from the Outside World.

### Close of the King's Tour.

THE reception of the King in Paris was most enthusiastic. On his arrival on Friday at the station he was received by President Loubet and the Ministers, and drove with the President in a carriage to the British Embassy. He was heartily welcomed all along the route. In the evening he was present at a gala performance at the Théâtre Français. On Saturday morning he accompanied the President to Vincennes for the great military review, which was witnessed by an immense assemblage of people. After lunching at the British Embassy the King drove with the President to Longchamps for the special race meeting arranged in his honour. In the evening he dined at the Elysée, and in reply to the toast of his health, proposed by the President, said that he desired a *rapprochement* of the two countries in their common interest and in the still higher interest of peace and civilisation. Subsequently he was present, in company with the President, at a gala performance at the opera, where he had a remarkable reception. On Sunday morning the King attended Divine service in the Embassy Church on foot, and afterwards lunched with M. Delcassé at the Foreign Office. On his way to and from the Quai d'Orsay he was lustily cheered by the crowds in the streets. In the afternoon he planted a red chestnut tree in the garden of the Embassy to commemorate his visit to Paris. Among those who witnessed this interesting incident were the children of the British Schools and the inmates of the Victoria Home for old Englishwomen. On Monday morning the King left Paris, being accompanied to the railway station by the President, to whom he stated that the remembrance of the reception he had met with would never fade. He arrived at Cherbourg on Monday evening, landed on the quay, and afterwards had a dinner party on his own yacht. On Tuesday morning at 8 o'clock the King left Cherbourg, reaching Portsmouth in time for lunch, and proceeding to Victoria. There he was met by the Prince of Wales and other members of the family, and amid hearty cheers drove to Buckingham Palace.

### A Famous Traveller.

THE name of Paul du Chaillu is little known to the present generation, but in the sixties the sensational account of his travels in Equatorial Africa caused much comment. His father held a consular appointment on the West Coast of Africa, and his son was educated at a Jesuit institution in that country. There he acquired a knowledge of the language, and of the habits of the natives which afterwards stood him in good stead. In 1855 he penetrated into the interior of Africa, travelling on foot or in canoes unaccompanied by any white man, upwards of 8,000 miles in four years. His travels resulted in the re-discovery of the great anthropoid ape, the gorilla, mentioned by Carthaginian navigators, and not entirely unknown to modern science, but practically forgotten. He returned to New York, and wrote a book "Explorations and Adventures in Equatorial Africa" which provoked considerable controversy. He again started for the African interior in 1865, taking with him astronomical and other instruments. He met with several misfortunes, and ultimately lost everything but the journals containing the records of his travels. His second expedition, however, enabled him to confirm the accounts given by the ancients of a pygmy people inhabiting the African forests.

### Dante on the Stage.

AS produced at Drury Lane Theatre by Sir Henry Irving, "Dante" is not a dramatisation of incidents in the life of the Italian poet, but a series of exciting adventures woven round an imaginary Dante. The play has been written especially for Sir Henry by MM. Sardou and Moreau, and the part assigned to the actor is admirably suited to his methods, whilst his acting is equal in merit to the best he has done. The prologue takes place in Pisa, outside the tower where Count Ugolino, his sons and grandsons are being starved to death. Here Dante has a love scene with his mistress, Pia de Tolomei, wife of Nello. Their converse about their seven-year-old daughter Gemma is interrupted by the groans of the prisoners. Dante protests against the cruelty of the Archbishop who detains them, seizes the crozier, dashes it to the ground, is excommunicated, and cursing Pisa, shakes its dust from off his feet. In the next scene, twelve years later, Dante has ventured to Florence disguised as a monk, and traced his daughter to Francesca da Rimini's house. Fearing Nello is going to murder her, Dante reveals his parentage, with the result that the unfaithful wife is sent by Nello to his castle in the pestilential Maremma—where she dies of malaria, Dante soothing her last hours—and Gemma is despatched to a convent. The father ultimately follows her; they are pursued, and Dante is stabbed, but not dangerously; Gemma is rescued by her lover. Later, Dante, losing all trace of the young people, learns from Beatrice in a vision that only the spirit of Pia can tell him where to find them, travels to Purgatory, conducted by Virgil, to gain the desired information, and ultimately arrives at the Papal Palace in Avignon just in time to rescue Bernardino and Gemma from the clutches of the Inquisition. The curtain falls as Dante blesses the happy couple, whose troubles now seem over.

### The New Gallery.

THE sixteenth summer Exhibition, at the New Gallery, possesses four examples of the wonderful power still left to Mr. Watts, who this year attains his 86th birthday. Two of these are landscapes, and it is difficult to believe that one, "The Two Paths," has not been painted by a young man just rising to the height of his fame. The most remarkable of the quartette is "The Sower of the Systems," a picture exceedingly rich in colour, a dim majestic figure allowing the rivers of gold to flow from His hands, so that they gradually break up into gleaming stars and diffuse light around. Amongst other landscapes "The Edge of the Marsh," flower-bedecked, by Mr. C. W. Wyllie; "Thorverton Bridge," in the valley of the Exe, with its dainty greys and greens, by Mr. Parsons; and "The Miller's Meadow," by Mr. Alfred East, are all worthy of mention. Far above all the portraits—in which the gallery is extremely rich—stands the portrait of Mr. Whistler, by M. Jean Boldini, the well-known Italian artist who lives in Paris, where this masterpiece has already been exhibited. It is a wonderful picture, lifelike in its rugged veracity, striking in the combative fierceness of its expression, and it at once arrests the attention and the admiration of the passer-by. A second canvas from the same artist is "Portrait of a Lady."

### A Woman on the "Brighton Walk."

AN enormous crowd of people watched the start of a number of members of the Stock Exchange who had arranged to walk from London to Brighton last Friday. The first and second to arrive at their destination completed the distance of 52 miles in nine hours and a half. Mrs. Alice Yorke, a resident of Eastbourne, commenting on what she describes as "the fuss on the 'great walk,'" writes: "I did it in the day a year or two ago, without any preparation or training, and without any equipment but the good health resulting from an active life, without wager or prize, and without after-effects, even of a special fatigue, though I am the mother of a family."

### A Book and its Story.

MISS COLERIDGE ON CHARLOTTE YONGE.*

Miss Christabel Coleridge, in presenting the life and letters of Charlotte Yonge to the public, remarks that "the task, in one way, has been easy, for so consistent, so harmonious a life has surely never been described, and rarely been lived. No inconsistent nor disappointing record has, or ever can, leap to light where she is concerned." This may be so, and Miss Coleridge who writes of Miss Yonge as the friend and adviser of a lifetime, under whose sympathy and criticism her best literary work was produced, is well calculated to speak, but this very perfection as a point of attraction is one upon which readers may disagree, as tending to lessen the interest in the personality of so admirable and charming a writer as Miss Yonge.

She, personally, had an aversion to publicity in keeping with her retired life and the date to which the best of her writings belong. So there is little in the book itself to make a life's history, and the numberless letters which fill a greater part of the book are of a kind more likely to be interesting to the surviving members of Miss Yonge's family and coterie than to the public generally. Before looking at the correspondence, or the autobiography itself, one naturally, when there are portraits, turns to them, to find very often stimulating suggestions of a personality which is more silently convincing than any verbal testimony. Four portraits of Miss Yonge are given. The first, from a painting by Richmond, shows her as a girl of twenty. The face is strikingly handsome, with regular features, and the dark eyes have a singularly sweet, even arch expression, which, with the dark hair worn in ringlets, goes to make a very charming picture of early Victorian girlhood. A later one, taken at the age of 35, makes it difficult to realise that in the strong, intellectual face we have the one whose earlier portrait was marked chiefly by the grace and charm of early womanhood. Certainly the portraits suggest the consistent development of intellect and character on harmonious lines, for benevolence and strength of individuality continue the dominant characteristics of the last one, taken six years before Miss Yonge's death, at the age of 75.

The first part of her Life is taken up by autobiography, and is chiefly family history without any particular interest to outsiders. Miss Yonge was the daughter of William Crawley Yonge, an officer in the Army, and Frances Mary Bargus. She was born at Otterbourne in 1823. The influences of race and place are evident in the moulding of her character. We are told of a favourite quotation of hers. "Character is the joint product of nature and nurture." She had an intense love and reverence for the memory of her forefathers, with some reason, for they were "Cultivated, reasonable gentlemen, sound Churchmen, and excellent parish priests in an age when we are apt to think that all country squires spent their time in hunting and drinking, and all parsons were idle and self-indulgent. . . . It is satisfactory to find that Charlotte Yonge's grandfather used all the proceeds of his living in the service of the Church. Lord Seaton, her mother's step-brother and her cousin by marriage, continued through life to be her ideal of the virtuous and honourable soldier. He was her justification for the chivalrous and knightly characters which she loved to draw."

One knows how to those who have not read her books, the mere mention of Miss Yonge's novels is sufficient to suggest heroes and heroines of impossible goodness, or of hopeless insipidity. But her characters are drawn from real life, and she would never accept the verdict of friends when they pronounced them "Too good to be true." "In her own environment she found those who were as good, and better than the heroes of her stories. She believed in good men and women because those whom she belonged to were good, and her mother's life, her talents and her education, had a great influence on Charlotte's early years. Family connections were life-long friends, and friendship with her was sweetened and strengthened by a drop of kindred blood." It is not possible to understand her life without realising how strong was this sense of kinship. As an only daughter Miss Yonge was the companion of her parents. Her girlhood, spent, as was her whole life, amid country scenes, was uneventful, but not without unusual advantages in the friends by whom she was surrounded.

Sir John Coleridge, Lord Seaton, Sir William Heathcote, Dr. Moberly, and many other distinguished men were among the more intimate. To Charlotte Yonge the inspiration necessary to the production of her stories had its source from within that happy home of which she was a fortunate member. We read: "This fact was the keynote of her character and produced that atmosphere of mingled ardour and submission in which she lived all her life, while all other contemporary and contending aspirations were so entirely outside her ken that she did not so much oppose them as remain in ignorance almost of their existence, and certainly of their force. It would be useless to deny that this environment produced limitations when in her turn she became the leader and the oracle, but it is also true that it made *her*, for which her generation may well be thankful to it."

In 1835, when Miss Yonge was twenty-two, John Keble was appointed vicar of Hursley. From that date began, what she acknowledged to be, "the great influence" of her life. "The traditions of the Yonge family were evangelical in tone, and their deep seriousness and profound value for scriptural knowledge fitted in with the lofty enthusiasms for the newly discovered, or newly accentuated, truths, which filled the young men and women of that generation with ardent zeal. Nothing could have been more alien from the minds of the 'Tractarians' than the idea that they invented doctrines or imposed them from abroad upon the Church of England. Their aim was to bring out what was already there, to develop what had been neglected or forgotten." In Miss Yonge, described at this time as "a vehement, eager, unformed girl," he had a loyal and exceptional disciple. From the age of twenty until the close of her life, Miss Yonge's leisure was devoted to literature. She had early formed the habit of writing down conversations of interest in which, from time to time, she had taken part; and she was a rapid writer, with unusual gifts of concentration and detachment. She frequently wrote a page of a story, a cameo, a letter, at the same time, leaving each one to dry, while she wrote the next. Her name will always be associated with the editorship of the "Monthly Packet." In 1853 she brought out "The Heir of Redclyffe," followed by "The Daisy Chain." The reception of the former work astonished author and publishers alike. So congenial was the hero Guy to the younger generation, that we are told that nearly all the young officers in her brother's regiment had a copy, and that he was taken as a Bild, or hero model, by the young pre-Raphaelites William Morris and Dante Rossetti. The part proceeds from the sale of "The Heir of Redclyffe" were given to the Melanesian Mission, and all the profits of "The Daisy Chain" to missions also. In spite of Miss Coleridge's avowal that her task has been easy, everyone after reading her life of Miss Yonge must feel that it has been otherwise, and she has succeeded in applying to the best advantage the scanty materials out of which to form her interesting memoir.

* "Charlotte M. Yonge: Her Life and Letters." By Christabel Coleridge. (1 vol. 10s. 6d. Macmillans.)

### For Reading to the Sick.

### PERFECT THROUGH SUFFERING.

Years and years and years my Master
Has been toiling for my good,
He has laid His Hand upon me;
Not His Hand alone, His Rood,
Proving, testing, and reproving,
Though I still misunderstood.

If to-day the skies are clearer
And the sunshine blazes bright,
While I seem a little nearer
To the throng that walk in white,—
Thou it is Who dwellest in me,
O my Love, and my Delight!

Bless me, let me be a blessing,
My exceeding great reward
To be living in Thy Presence,
Always, ever with the Lord,
In Thee, for Thee, to Thy Glory,
And according to Thy Word!

*Anon.*

The connection between a life of suffering and the fellowship with Christ is so close that it is impossible to study the one apart from the other.

When the early followers of the Saviour were called upon to follow Him and to preach the Gospel, they were plainly and boldly told of all the trials and difficulties which should meet them. For awhile the disciples seemed only capable of understanding the bare outline both of their mission and their reward; but gradually, by the teaching of the Holy Spirit, they became in a measure conscious that through their sufferings they were to glorify God. In the maturer teaching of the Epistles this glorifying is presented in a clear and twofold aspect to all Christians. St. Peter in his old age dwells emphatically upon it—firstly, as being the high privilege of the disciple of Christ that he should be permitted to glorify God through suffering; and, secondly, that in his sufferings God will give him the "spirit of glory:" it shall "rest" upon him.

Here is not only the opening out of illimitable vistas of service and honour for ever and ever, but the very highest encouragement for the present. Now, at this time, Christ is to be glorified, and now also will the spirit of glory rest or abide on the humblest of His followers who is "a partaker of Christ's sufferings."—*Mrs. C. Campbell.*

Thou, that to Thy Divinity
A human nature didst ally
By mortal birth.

And in that form didst suffer here
Torment and agony and fear
So patiently.

By Thy redeeming grace alone
And not by merits of my own
O, pardon me.—*Longfellow.*

Show me the path! I had forgotten Thee
When I was happy and free,
Walking down here in the gladsome light of the sun;
But now I come and mourn; oh set my feet
In the road to Thy blest seat!
And for the rest, O God, Thy Will be done!

*Jean Ingelow.*

### Notes and Queries.

### REGULATIONS.

**The Editor is always willing to answer in this column, without any fee, all reasonable questions, as soon as possible.**

**But the following rules must be carefully observed:—**

**1. Every communication must be accompanied by e name and address of the writer.**

**2. The question must always bear upon nursing, directly or indirectly.**

**If an answer is required by letter a fee of half-a-crown must be enclosed with the note containing the inquiry. We cannot undertake to forward letters addressed to correspondents making inquiries unless a stamped envelope is enclosed.**

### *Special.*

(56) In reply to *Nurse S.* and several other correspondents who write to inquire why their questions have not yet been answered, we may state that questions can only be answered in their turn, which, owing to the great number received, often does not come for several weeks. Correspondents who require an immediate reply by post must comply with the regulations, and enclose a fee of half a crown.

### *Algiers.*

(57) The Matron of the British Cottage Hospital, Alger Mustapha, Algiers, invites *Nurse G.*, who inquired for a nursing post in Algiers, to apply to her.

### *Solution for Soaking Sheets.*

(58) I should like to know the best solution for soaking sheets etc. from typhoid patients. I use 1 in 1000 perchloride of mercury, but find it often turns the things black in washing and no amount of boiling will get out the stains.—*An Australian Matron.*

Perchloride of mercury, even as weak as 1 in 2,000, will stain linen irretrievably. Carbolic solution 1 in 20 will answer all practical purposes of disinfection without injuring the linen in any way, and you will probably find it quite satisfactory.

### *Lectures.*

(59) Will you let me know if there is an inexpensive book giving good hints, or a short course of lectures, on elementary nursing suitable for working women?—*Nurse A.*

Write to the Secretary of the National Health Society, 53 Berners Street, W.

### *Dressing.*

(60) I should like to know what is the best dressing to apply to the umbilical cord of a newly-born infant, and if lint, dipped in spirits of wine, is advisable; also if the first dressing should remain undisturbed for two days.—*Novice.*

You must follow the instructions of the medical man in charge of the case.

### *Maternity.*

(61) I wish to train as monthly nurse. I am 37, and have good health. Will you kindly tell me which is the best hospital at which to train, and of a book which would help me before training?—*Eunice.*

Queen Charlotte's Hospital; the British Lying-in Hospital Endell Street, St. Giles', W.C. (age up to 40, fees £22 10s.); the City of London Lying-in Hospital, City Road, E.C. (fees £21); the Clapham Maternity Hospital, Jeffrey's Road, Clapham, S.W. (age to 40, fees £27); the East End Mothers' Home, 394 and 396 Commercial Road, E., (£15 15s.); and the General Lying-in Hospital, York Road, Lambeth, S.E. (fees £26 5s.) All these hospitals and schools prepare for the certificate of the London Obstetrical Society. "Practical Handbook of Midwifery," Haultain. Scientific Press, 28 Southampton Street, Strand, W.C.

### *Urinal.*

(62) Can you tell me if there is any new urinal appliance, rubber preferred, which an elderly helpless male patient could use in bed? He is badly ruptured.—*A. B.*

Any surgical instrument maker would be able to supply you.

**Standard Nursing Manuals.**

"The Nursing Profession: How and Where to Train." 2s. net; 2s. 4d. post free.

"Nursing: Its Theory and Practice." (Revised Edition). 3s. 6d. post free.

"Surgical Ward Work and Nursing." (Revised Edition). 3s. 6d. net.; 3s. 10s. post free.

"Practical Handbook of Midwifery." (New Edition). 6s. net; 6s. 3d. post free.

"Notes on Pharmacy and Dispensing for Nurses." 1s. post free.

"Fevers and Infectious Diseases." 1s. post free.

"The Art of Massage." (New Edition). 6s. post free.

